# Sculpting Success: The Importance of Diet and Physical Activity to Support Skeletal Muscle Health during Weight Loss with New Generation Anti-Obesity Medications

**DOI:** 10.1016/j.cdnut.2024.104486

**Published:** 2024-10-18

**Authors:** Gregory J Grosicki, Nikhil V Dhurandhar, Jessica L Unick, Shawn M Arent, J Graham Thomas, Holly Lofton, Madelyn C Shepherd, Jessica Kiel, Christopher Coleman, Satya S Jonnalagadda

**Affiliations:** 1 Department of Scientific and Clinical Affairs, Medifast, Inc, Baltimore, MD, United States; 2 Department of Nutritional Sciences, Texas Tech University, Lubbock, TX, United States; 3 The Miriam Hospital Weight Control and Diabetes Research Center, Providence, RI, United States; 4 Warren Alpert Medical School at Brown University, Providence, RI, United States; 5 Department of Exercise Science, University of South Carolina, Columbia, SC, United States; 6 New York University Grossman School of Medicine, New York, NY, United States

**Keywords:** GLP-1 receptor agonists, nutrient-stimulated hormone-based therapy, muscle mass, protein, exercise, lifestyle modification, GIP (glucose-dependent insulinotropic polypeptide), fat-free mass

## Abstract

Obesity is a public health crisis, with prevalence rates tripling over the past 60 y. Although lifestyle modifications, such as diet and physical activity, remain the first-line treatments, recent anti-obesity medications (AOMs) have been shown to achieve greater reductions in body weight and fat mass. However, AOMs also reduce fat-free mass, including skeletal muscle, which has been demonstrated to account for 20% to 50% of total weight loss. This can equate to ∼6 kg or 10% of total lean mass after 12–18 mo, a loss comparable to a decade of human aging. Despite questions surrounding the clinical relevance of weight loss-induced muscle loss, the importance of adopting lifestyle behaviors such as eating a protein-rich diet and incorporating regular resistance training to support skeletal muscle health, long-term weight loss maintenance, and overall well-being among AOM users should be encouraged. Herein, we provide a rationale for the clinical significance of minimizing weight-loss-induced lean mass loss and emphasize the integration of diet and physical activity into AOM clinical care. Owing to a lack of published findings on diet and physical activity supporting skeletal muscle health with AOMs, specifically, we lean on findings from large-scale clinical weight loss and diet and exercise trials to draw evidence-based recommendations for strategies to protect skeletal muscle. We conclude by identifying gaps in the literature and emphasizing the need for future experimental research to optimize skeletal muscle and whole-body health through a balance of pharmacotherapy and healthy habits.

## Introduction

The worldwide prevalence of obesity has nearly tripled since 1975, reaching pandemic levels [1]. In the United States, >2-in-5 adults live with obesity [2], increasing their risk of numerous chronic health conditions and premature death [3]. To improve obesity-related complications, a weight loss of 5–15% is recommended, with greater benefits observed with larger weight reductions [4]. Lifestyle modifications, such as diet and physical activity, are the first-line treatment for overweight and obesity, resulting in an mean 1-y weight loss of ∼5–10% of initial body weight and significant reductions in chronic disease risk [5]. However, many individuals fail to achieve clinically significant weight loss with lifestyle modification alone [6,7].

New generation anti-obesity medications (AOMs), particularly nutrient-stimulated hormone (NuSH)-based therapies such as glucagon-like peptide-1 receptor agonists (GLP-1 RAs), show promise in addressing the obesity pandemic. NuSH-based therapies promote weight loss primarily by inducing satiety and delaying gastric emptying, leading to weight loss that often surpasses that seen with lifestyle modification alone [8,9]. Seminal AOM clinical trials demonstrated that weekly injections of both semaglutide and tirzepatide as an adjunct to lifestyle intervention resulted in average body weight reductions of ∼15–25%, with over 85% of participants achieving clinically relevant weight loss (i.e., ≥5%) in 18 mo or less [8,9]. However, AOMs reduce both fat and fat-free mass (FFM), including skeletal muscle, one of the most dynamic and plastic tissues in humans body that contributes significantly to numerous bodily functions (e.g., mechanical and metabolic) [10]. A review of the effects of GLP-1 RAs and sodium-glucose cotransporter 2 inhibitors reported that the proportion of lean body mass reduction ranges between 20–50% of total weight loss [11]. This can equate to ∼6kg or 10% of total lean mass after 12–18 mo of treatment [9], which is comparable to the muscle loss that typically occurs over the course of a decade of human aging [12,13].

Given the recent Food and Drug Administration approval of new generation AOMs for chronic weight management in adults with overweight or obesity, herein we emphasize the importance of incorporating lifestyle modification into AOM-assisted weight loss to support skeletal muscle health in this growing patient population. By applying this knowledge clinically, a large and growing number of AOM users could be better supported through the development of evidence-based lifestyle strategies to support skeletal muscle health, long-term weight loss maintenance, and overall health and well-being.

### A case for the clinical relevance of weight-loss-induced muscle mass loss

Skeletal muscle comprises ∼40% of total body mass and plays a vital role in overall health and well-being [10]. As reviewed in detail elsewhere [14], skeletal muscle is best known for its capacity to generate force and power, which are crucial for the ability to perform activities of daily living and may be compromised by diet-induced weight loss [15]. Because weight loss-associated declines in fat mass typically exceed reductions in lean mass, the functional implications of this phenomenon have been questioned on the basis of previous associations between higher FFM to fat mass ratios and better physical function (e.g., balance testing and walking speed) [16]. However, there is a paucity of data to confirm the functional and other systemic consequences of AOM-mediated muscle loss. Furthermore, the importance of skeletal muscle extends far beyond its mechanical functions [17]. It plays essential roles in whole-body protein metabolism and energy expenditure, which are often less appreciated but equally important [14].

In terms of protein metabolism, skeletal muscle helps to replenish and maintain circulating amino acids, which are taken up by other tissues to serve as precursors for the synthesis of proteins and hepatic gluconeogenesis [18,19]. Additionally, skeletal muscle metabolism is the most adaptable component of resting metabolic rate [20], which is the largest determinant of total daily energy expenditure [21]. Therefore, maintaining muscle mass is vital to sustaining a higher metabolic rate, which not only increases total daily energy expenditure but is also linked to improvements in metabolic flexibility [22] - the ability of an organism to respond or adapt to changes in metabolic demand through transitions in fuel selection [23]. For NuSH-based therapies, such as semaglutide, where an average weight regain of two-thirds of the previous weight loss is typically observed within 1 y of medication cessation [24], preserving muscle mass to maintain metabolic rate and flexibility and optimize physical function, may be critical.

More broadly, reductions in skeletal muscle mass, such as those that occur with aging (i.e., sarcopenia) [25], are associated with chronic diseases (e.g., cardiovascular disease and cancer) and are predictive of survival [26,27]. This is particularly relevant for AOM users, who are typically 50–60 y of age [28]; this is a period of life marked by increased vulnerability to age-associated muscle loss, mobility deficits, and loss of functional independence [12]. Thus, when evaluating the clinical relevance of weight-loss-induced muscle loss, it is imperative to consider the diverse and critical roles that skeletal muscle plays in supporting many facets of health. In this context, we believe there is a rationale for the clinical significance of minimizing weight-loss-induced skeletal muscle loss and a need to focus on preservation strategies.

### Lifestyle behaviors to support muscle health during weight loss

When the Food and Drug Administration approved the new generation of AOMs for weight management, it was specified that these drugs be used in combination with “a reduced calorie diet and increased physical activity” [29]. However, due to the novelty of these medications, there is a lack of evidence regarding the impact of lifestyle strategies that support muscle health among AOM users. Therefore, below, we rely on data from systematic reviews and meta-analyses, as well as large-scale weight loss and diet and exercise trials, for evidence-based muscle preservation strategies. We focus on dietary protein and resistance training (RT) as key driving forces of muscle protein synthesis (MPS) [30] with the potential to attenuate weight loss-induced muscle loss in persons with overweight or obesity.

### Dietary protein to support skeletal muscle during weight loss

#### Dietary protein and its role in weight loss

High-quality protein is an essential component of a healthy diet, playing a key role in building and repairing tissues, producing enzymes and hormones, and supporting overall growth and development. For inactive but healthy adults, the recommended dietary allowance for protein intake is 0.8 g/kg body weight per day (g/kg/d). However, this recommendation is primarily based on nitrogen-balance studies and defines minimum protein intake requirements, which have several limitations and may not address the diverse and dynamic needs of the entire population [31]. An individual’s recommended protein intake can vary based on several factors, including activity level, age, sex, weight, caloric intake, and overall health [32]. For instance, those aiming to build muscle mass and/or individuals focusing on preserving skeletal muscle during weight loss have higher protein intake requirements [33,34].

In the context of weight loss, meta-analyses have demonstrated that increased protein intake (i.e., greater than the recommended dietary allowance) helps to reduce body weight and improve body composition by decreasing fat mass while preserving FFM in both younger [35] and older [36] individuals. These benefits may result from enhanced satiety, increased thermogenesis, and/or the provision of additional essential amino acids (EAAs) for MPS [33,37,38]. Consequently, professional nutrition and obesity societies recommend higher protein intake as part of effective weight loss strategies to optimize body composition and support long-term weight loss maintenance [39], as described in greater detail below.

#### Strategies for determining daily protein intake recommendations

Table 1 [34,[40], [41], [42]] provides a comparison of 4 protein intake strategies to support healthy weight loss and management. Commonly, consuming 1.0–1.5 g/kg of actual body weight per day is advised [40]. A meta-regression by Krieger et al. [43] demonstrated that FFM retention during weight loss was increased by protein intake in a dose-response manner, up to 1.2 g/kg actual body weight per day. This threshold has since been replicated by experimental research seeking to identify the optimal cut-point for protein intake based on muscle mass accretion during weight loss [44]. However, a limitation of these studies is that they did not account for the impact of physical activity on protein requirements. The International Society of Sports Nutrition recommends a range of 1.4-2.0 g protein/kg actual body weight per day for exercising individuals [34], and this higher range may be appropriate for AOM users.

**TABLE 1:** Comparison of protein intake strategies to support healthy weight loss and management: recommendations, benefits, and limitations.

Strategy	Recommendations	Benefits	Limitations
Relative to actual body weight	1.0–1.5 (General population) g/kg actual body weight per day (Muscogiuri et al. [40]) 1.4–2.0 (Exercising individuals) g/kg actual body weight per day (Jäger et al. [34] 2017)	• Relatively straightforward • Supported by extensive research	• May overestimate protein needs for individuals with overweight or obesity • Does not account for variations in body composition
Relative to fat-free mass	≥1.5 g/kg fat-free mass per day (Dekker et al. [41] 2022)	• More personalized to reflect actual protein needs • May better support lean mass preservation	• Requires accurate body composition measurement • Lack of extensive research to confirm recommendations per efficacy
Relative to ideal body weight	1.3–1.5 g/kg ideal body weight per day (Singer et al. [42] 2009)	• Reduces the chance of excess protein intake recommendations in individuals with higher fat mass	• Ideal body weight can be subjective • Does not account for variations in body composition • May underestimate protein needs in some individuals
Absolute quantity	total g range per day (e.g., 80–120)	• Simple and easy to understand • Can promote adequate protein intake across varying body weights • Encourages consistency	• Recommendation requires further research • May not be precise for all individuals, especially those with very low or very high body weight • Could lead to inadequate or excessive protein intake recommendations in some cases

Alternatively, it has been suggested that protein intake recommendations be based on FFM rather than total body weight, which may be more reflective of true interindividual protein requirements [45,46]. For example, protein intake recommendations based on body weight may exceed FFM requirements for individuals who are overweight or obese. If body composition data are available, protein intakes of ≥1.5 g/kg FFM per day are recommended [41], though more research is needed to confirm the efficacy of this approach. It has also been proposed that protein intake recommendations in individuals with an elevated BMI (in kg/m^2^) be made relative to an “ideal body weight” (e.g., weight where BMI = 27.5) to temper excessive protein intake recommendations that may result from guidance based on actual body weight [41]. In such a case, 1.3–1.5 g/kg ideal body weight per day may be considered, though AOM-specific data are lacking [42].

Given the complexities in determining ideal protein intake based on body composition or ideal body weight, alternative simplified approaches include recommending protein intake by macronutrient distribution (e.g., >25% of total calories) [47] or an absolute daily quantity (e.g., 80–120 g/d). A unique strength of the absolute daily quantity approach is that it ensures a greater relative protein intake (g/kg actual body weight per day) in individuals with a lower body weight and a relatively lower intake in those with a higher body weight, potentially aligning more closely with their FFM needs. The simplicity of this recommendation may also enhance adherence through a clear and attainable goal.

In sum, the available evidence appears to support that individuals taking new generation AOMs aim for ≥1.2 g/kg actual body weight per day, but we acknowledge that interindividual variability exists and that there is a need for more population-specific data to confirm this recommendation. Additionally, it’s important to consider that physically active individuals may have increased protein requirements. For practical purposes, an absolute protein intake recommendation may help to ensure sufficient protein intake with body weight changes across different individuals, but rigorous studies to confirm the theoretical benefits of this approach are needed.

#### Practical considerations: protein quality, timing, and distribution

The muscle-sparing benefits of higher dietary protein during weight loss have been observed in numerous, but not all, studies [35,48,49]. This inconsistency can be attributed to differences in dietary compliance [33] and study design methods, including sample size, duration, participant characteristics, and macronutrient composition of the prescribed diets [35]. For instance, study durations of <12 wk are likely inadequate to detect meaningful skeletal muscle protective (i.e., myoprotective) benefits, which may be explained by minimal and insufficient lean mass reductions in the control group early in treatment [35]. Additionally, certain conditions such as hyperinsulinemia [50] might interfere with MPS, rendering increased protein intake alone insufficient to preserve FFM during weight loss. This may be particularly important for contemporary AOM users, many of whom have type 2 diabetes. For these individuals, complementary anabolic strategies, such as RT (see *RT to* *support* *skeletal muscle during weight loss*), should be considered. Notably, a study by Magkos et al. [48] found that increased protein intake failed to attenuate reductions in lean mass in adults with overweight or obesity who were following a very low-calorie diet (<800 kcal/d) [48]. This finding alludes to the idea of a minimum caloric threshold below which the myoprotective benefits of increased protein intake are diminished and supports the value of determining and maintaining caloric intake above this threshold.

Variability in outcomes may also stem from differences in protein feeding patterns (e.g., timing and distribution) and/or protein quality per type. Regarding timing, consumption of EAAs before, during, or immediately after exercise has been shown to enhance the anabolic response by potentiating intracellular signaling and MPS [[51], [52], [53], [54]]. Greater MPS rates were also reported when protein intake was distributed evenly throughout the day (i.e., 4 x 20 g servings every 3 h) compared to less frequent higher volume servings (2 x 40 g every 6 h) [55], as is seen in the typical American dietary pattern where protein intake is heavily skewed toward the evening with a large meal [56].

A meta-analysis by Schoenfeld et al. [57] showed a small to moderate effect of protein timing surrounding RT on muscle hypertrophy. However, this effect was attenuated following covariate adjustment (difference = 0.16 ± 0.11; confidence interval: –0.07, 0.38; *P* = 0.18), with total protein intake emerging as the strongest predictor of muscle growth [57]. Although this observation refutes the idea that protein timing is critical to muscular adaptation, context is important – included studies almost exclusively consisted of young, healthy, normal-weight individuals and only 3 of 20 studies matched protein intake between experimental (i.e., timed protein intake) and control groups. Furthermore, very few of the studies actually manipulated protein timing. Last, the effect of protein timing on muscular adaptation may be obfuscated by the profound anabolic stimulus of RT. Nonetheless, the importance of daily total protein intake as the greatest determinant of muscular adaptation is clear. In the context of NuSH-based therapy-mediated appetite suppression where *ad libitum* energy intake is reduced ∼35% (∼415 kcal compared with ∼640 kcal for lunch) [58], protein timing strategies that maximize daily protein intake (e.g., small frequent doses throughout the day) may confer the greatest myoprotective benefits.

There is also evidence that protein type (i.e., source) has a significant effect on whole-body protein anabolism [59]. The overall quality of a dietary protein can be evaluated by its digestibility and amino acid profile [60,61]. In general, animal, dairy, and soy-based proteins contain the highest percentages of EAAs and result in greater hypertrophy and protein synthesis when compared with incomplete protein-matched controls (e.g., nuts and seeds), which have poorer digestibility and lower relative EAA content [34,62]. A recent study by Lim et al. [63] compared aminoacidemia and MPS responses to an incomplete pea-based protein with and without added leucine to a conventional whey protein isolate (i.e., high-quality dairy-based protein). As anticipated, MPS responses to whey exceeded those with pea-based protein. However, the addition of the EAA leucine, which is the key EAA that stimulates MPS [52], significantly increased MPS with pea-based protein to achieve levels comparable to those with whey. Although AOM-specific data are lacking, it seems logical to “make every bite count” by focusing not only on protein quantity but also quality. To this end, a focus on high-quality protein sources rich in EAAs, such as animal and dairy-based proteins, or certain high-quality plant-based proteins, like soy, that will ensure ∼2–3 g of leucine is consumed per feeding to stimulate the protein translational machinery is recommended [34].

#### Dietary protein - summary and recommendations

To summarize, increased protein intake appears to be important for preserving FFM during weight loss. Based on existing evidence, a minimum total protein intake of 1.2 g/kg actual body weight per day in the absence of an extreme caloric deficit (i.e., a very low-calorie diet) is recommended. This protein intake should ideally be evenly distributed throughout the day, with a focus on high-quality protein sources rich in EAAs (e.g., 10 g per feeding), including leucine (e.g., 2–3 g per feeding), to support MPS [64]. It is also important to note that protein needs may be higher (1.4–2.0 g/kg actual body weight per day) for individuals who engage in regular physical activity [34].

### RT to support skeletal muscle during weight loss

#### The importance of physical activity in weight loss

The importance of physical activity [65], including both aerobic exercise and RT [13,66], to optimize changes in body composition with weight loss has ample support in the scientific literature. First, physical activity may reduce body weight and adiposity beyond what is achieved through energy restriction alone, and there appears to be a dose-response relation between exercise volume and the magnitude of weight loss observed [67]. A 6-mo behavioral weight loss intervention of young adults with overweight or obesity demonstrated that moderate-vigorous physical activity completed in bouts of ≥10 min was an independent predictor of weight loss (β = –0.21) [68]. Similar findings were reported by Lundgren et al. [69] in sedentary participants taking the GLP-1 RA medication liraglutide, half of whom participated in a heart rate-monitored aerobic exercise intervention designed to meet WHO specifications (i.e., 150-min moderate-intensity activity or 75-min vigorous-intensity, or an equivalent combination of both per week) [70]. Combining exercise with liraglutide resulted in a greater weight loss than exercise alone (difference: –5.4 kg; confidence interval: –9.0, –1.7; *P* = 0.004) and a reduction in body fat percentage (3.9% points) that was approximately twice as much as exercise (–1.7% points) or medication (–1.9% points) alone. Dual-energy x-ray absorptiometry-derived lean mass was also retained in the combination group, suggesting that even aerobic exercise may help to spare lean mass during AOM-mediated weight loss. However, lean mass was also maintained in the liraglutide-only group. Other NuSH-based therapies, such as semaglutide and tirzepatide, tend to have a greater impact on reducing body weight [8,9], and thus this preservation of lean mass through aerobic exercise observed in other contexts may not generalize.

#### Comparing aerobic and RT

Regarding activity type, a randomized trial by Willis et al. [71] compared the effects of aerobic and RT on body mass, fat mass, and lean mass in middle-aged adults who have overweight or obesity. Total body mass and fat mass were reduced to a greater extent with aerobic training alone (–1.8 ± 3.0 kg) and when combined with RT (–1.6 ± 3.2 kg), compared with RT alone (–0.8 ± 2.3 kg). Meanwhile, RT alone (+1.1 ± 1.5 kg) and, when combined with aerobic training (+0.8 ± 1.4 kg), increased lean body mass more than aerobic training alone (–0.1 ± 1.2 kg). The authors concluded that aerobic training is the optimal mode for reducing fat and body mass, whereas a program including RT is needed for increasing lean mass in this population. Importantly, these benefits can be achieved through opposing exercise modes. For instance, in adults with visceral adiposity, aerobic exercise attenuated the loss of skeletal muscle during 12 wk of energy restriction [72]. Likewise, a systematic review and meta-analysis demonstrated that combining RT with caloric restriction was most effective for reducing body fat percentage and whole-body fat mass in individuals with overweight and obesity across the lifespan [73]. Ideally, combining aerobic and RT during energy restriction would maximize the benefits of both exercise modes [74]. However, if time is limited, the available evidence appears to suggest that RT should be prioritized to best support lean mass during weight loss among AOM users [75,76], as described in greater detail below.

#### Prioritizing RT for lean mass preservation

As with nutritional intake, there are several variables to consider when designing exercise programs seeking to optimize health during weight loss. Clinical guidelines recommend between 150 and 250 min/wk of moderate-intensity physical activity to improve weight loss with moderate dietary restriction, but note that greater benefits are observed with durations of over 250 min/wk [74]. Meanwhile, the physical activity guidelines for Americans recommend that RT targeting all major muscle groups should be performed at least twice-a-week [77]. However, even a single weekly RT session with loads of 50–75% of maximal strength may be sufficient to improve muscle size, strength, and power in untrained older individuals [78,79]. Meanwhile, in untrained older females, 2 sessions per week of RT was found to be as beneficial as 3, provided that the number of sets performed was equal (i.e., 6 total sets per week) [80]. Though the translation of these findings to supporting skeletal muscle with AOM-mediated weight loss is uncertain, it seems pragmatic to advocate for the value of at least twice-a-week RT to support skeletal muscle health, with training sessions performed once-a-week still likely to provide some initial benefit.

#### Guidelines for prescribing RT

Concerning the design of these RT sessions, to increase muscle mass, it is generally recommended that individuals perform 1–3 sets of 8–12 repetitions per set, using an intensity >60% of 1-repetition maximum (1-RM) (i.e., the maximum amount of weight that can be lifted with proper technique for only 1 repetition for a specific exercise [81]), with 8–10 exercises per session [82]. As is customary with any training program, a stepwise approach that gradually progresses in sets, repetitions, and training load to minimize excessive soreness and injury risk is advised [83]. Alternative muscle-strengthening approaches that do not require expensive equipment or supervision from qualified professionals are also worthy of consideration. For instance, a pilot study from Normandin et al. [84] showed that daily weighted vest use during weight loss is a feasible and safe approach to preserving leg muscle power in well-functioning older adults with obesity. Though not a weight loss study, Cintineo et al. [85] recently determined that an RT program comprised of portable, minimal equipment such as sandbags, resistance bands, and suspension systems was effective at improving body composition and strength in military personnel. Although larger and longer-duration studies featuring AOM users are needed, these low-cost and more accessible approaches are appealing.

#### RT – summary and recommendations

To summarize, although aerobic training appears to be the optimal mode of exercise for reducing fat mass and body mass, a program including RT seems to be important for preserving FFM during weight loss. Evidence suggests prioritizing RT at least twice-a-week, performing 1–3 sets of 8–12 repetitions for each major muscle group to optimize muscle health and support FFM retention.

### Synergistic effects of dietary protein and RT to support skeletal muscle during weight loss

#### The synergy between dietary protein and RT

Increased dietary protein intake and RT independently benefit skeletal muscle during weight loss. However, there is compelling evidence for a synergy between these behaviors. For instance, a single bout of RT (8 sets of 8 repetitions at 80% 1 RM) in the postabsorptive state stimulates MPS by >100% [86], but combining RT with protein feeding elicits additive benefits for acute MPS in healthy untrained male [87]. This synergy is supported by a systematic review and meta-analysis demonstrating the superiority of combined exercise training (both aerobic and RT) and a high protein diet (i.e., >0.8 g/kg/d or 20% or more of caloric intake through protein) over exercise alone in preserving FFM during weight loss in adults with obesity [88]. Similarly, another systematic review and meta-analysis of nonweight loss studies in older adults demonstrated that combining RT with protein supplementation (i.e., any interventions containing leucine, whey, casein, lean meat, low-fat milk, or a related mixture) is more effective than RT alone in enhancing muscle mass and strength in the absence of intentional negative energy balance [89]. These findings collectively support the idea that complementing RT with increased protein intake will help to support skeletal muscle health during weight loss better than RT alone in AOM users.

Adding RT to increased dietary protein during weight loss also yields superior myoprotective benefits than protein intake in isolation. The Layman et al. [90] 2005 study provided early evidence for this benefit. Females with overweight or obesity were placed on an energy-restricted diet and randomized to either a high protein (1.6 g/kg/d protein; 1.5 carbohydrates per protein ratio) or high carbohydrate (CHO) (0.8 g/kg/d protein; 3.5 carbohydrates per protein ratio) group, with or without exercise (5 d/wk walking and 2 d/wk RT). Participants in the protein-rich diet PRO and protein-rich diet plus exercise (PRO+EX) groups lost more total weight (PRO: –8.7 and PRO+EX: –9.8 kg) and fat mass (PRO: –5.9 and PRO+EX: –8.8 kg) and tended to lose less lean mass (PRO: –2.0 and PRO+EX: –0.4 kg), than those in the [CHO and carbohydrate plus exercise (CHO+EX)] groups (weight: CHO: –7.8 and CHO+EX: –6.7 kg; fat mass: CHO: –5.0 and CHO+EX: –5.5 kg; lean mass: CHO: –2.7 and CHO+EX: –1.0 kg); the greatest reductions in body fat and smallest changes in lean mass were observed in the PRO+EX group. In another study, physically active young adults with a BMI between 22–29 consuming 1.2 g/kg actual body weight per day insufficiently preserved FFM during weight loss [91], but this was rescued by adding RT (6 d/wk) [92]. Meanwhile, doubling protein intake to 2.4 g/kg actual body weight per day, along with RT (6 d/wk), increased FFM (1.2 ± 1.0 kg) over 4 wk despite a marked energy deficit (40% reduction compared with requirements) and fat loss (–4.8 ± 1.6 kg).

#### A synergistic approach – summary and recommendations

**FIGURE 1:**
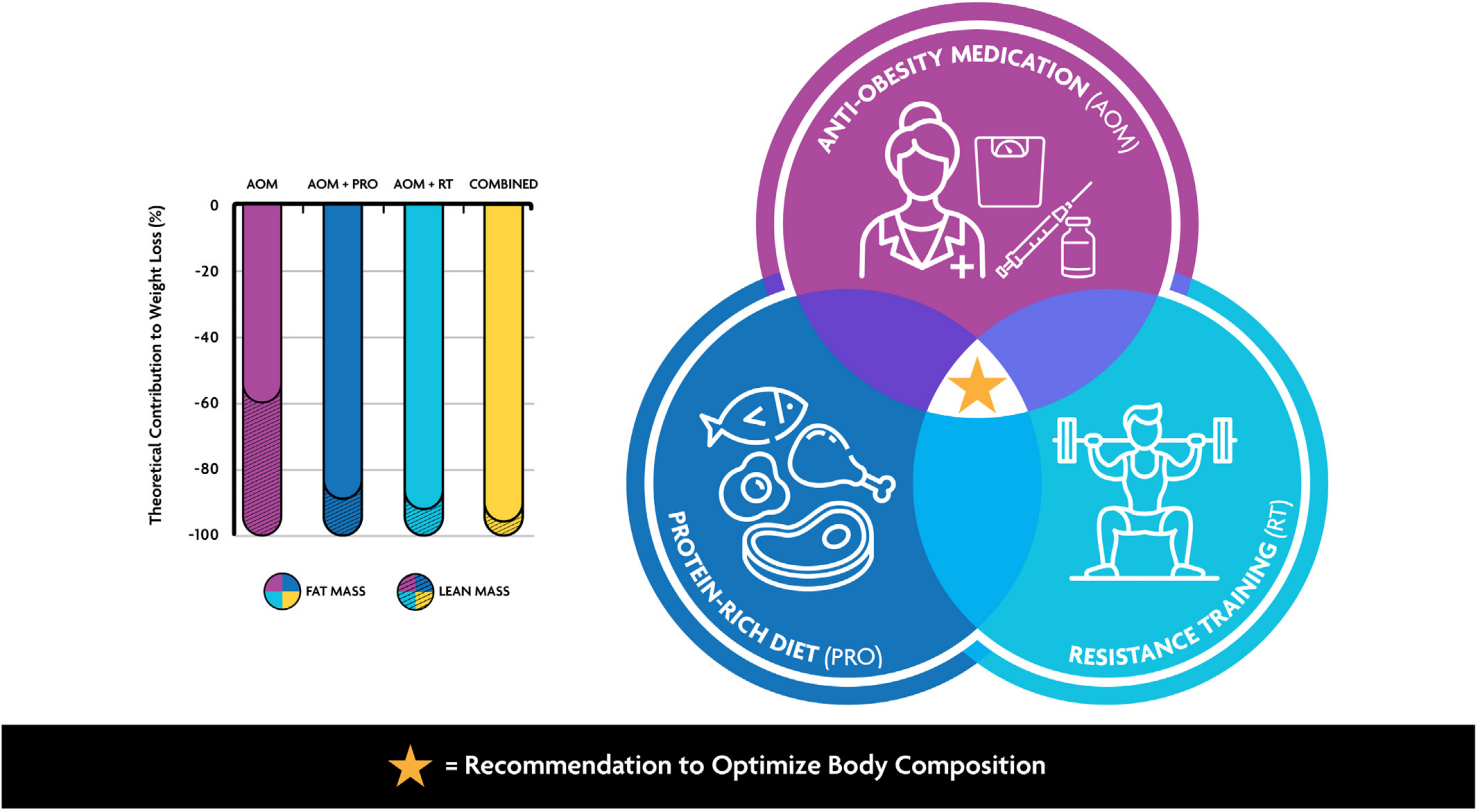
Illustration of an integrated approach consisting of pharmacotherapy, a protein-rich diet (PRO), and resistance training to optimize body composition among users of new generation anti-obesity medications (AOMs). The bar graph represents the theoretical relative contribution of fat mass (solid bars) and lean mass (striped bars) to weight loss under 4 conditions: AOM alone (pink), AOM plus PRO (AOM + PRO; dark blue), AOM plus resistance training (AOM + RT; light blue), and the combination of all 3 (AOM + PRO + RT; yellow). The relative contribution of lean body mass to total weight loss with AOMs is suggested to range between 20% and 50% [11]. The data for AOM alone (pink) are inferred from body composition changes measured using dual-energy x-ray absorptiometry in a double-blind trial of once-weekly semaglutide in adults with overweight or obesity [8]. Simulated data for AOM + PRO are based on meta-analyses demonstrating that increased protein intake improves body composition by decreasing fat mass while preserving lean mass during weight loss [35,36]. AOM + RT data are simulated from studies showing that combining liraglutide with exercise reduced body fat percentage twice as much as medication alone while preserving lean mass [69], as well as evidence supporting the role of resistance training in increasing lean body mass in adults with overweight or obesity [71]. Finally, the combined approach (AOM + PRO + RT) is simulated from original research [90] and systematic reviews [88], showing that the combination of high-protein diets and exercise training leads to the greatest reductions in body fat and lean loss of lean mass during weight loss.

Although the dose of RT and protein supplementation varied across studies, the above findings strongly support the synergistic benefits of combining high protein intake and RT to support skeletal muscle health during weight loss. These findings have important implications for clinical guidelines surrounding AOM use, suggesting that incorporating dietary protein and physical activity, particularly RT, is likely to enhance the effectiveness and sustainability of NuSH-based therapies (Figure 1) [8,11,35,36,69,71,90,88]. By promoting better preservation of FFM and optimizing body composition, this approach may not only improve weight loss outcomes but also support long-term weight loss maintenance and overall health and well-being for individuals using new generation AOMs.

### Research gaps and future directions

Although the current evidence supports the synergistic benefits of dietary protein and RT for preserving FFM during weight loss, more research is needed to evaluate their effectiveness in the context of heterogeneous populations of AOM users and relevant biological variables (e.g., age and sex). Clinical trials combining AOMs, RT, and dietary protein interventions are essential to determine the impact of these lifestyle behaviors on muscle preservation, as well as other health outcomes such as bone health [93], cardiovascular health [94], gastrointestinal health and gut microbiome [95], and psychological well-being [96]. Furthermore, it is important to assess the contribution of other lifestyle behaviors, such as hydration and sleep, to muscle and overall health outcomes in AOM users. Last, given known changes in food preferences and ingestive behaviors with NuSH-based therapies [97], we hypothesize that these medications may facilitate greater adoption of and long-term adherence to positive lifestyle changes by liberating cognitive resources previously focused on food. Along these lines, preclinical studies have identified dopaminergic effects of GLP-1 RAs with beneficial implications for reducing substance use disorders [98]. Additional research in these areas will provide a more holistic understanding of how to optimize health and well-being for individuals undergoing weight loss treatments with new generation AOMs.

In conclusion, the rising prevalence of obesity necessitates effective strategies for chronic body weight management. New generation AOMs show promise by significantly surpassing the magnitude of weight loss achieved through lifestyle modification alone. Accompanying this greater magnitude of weight loss will likely be a significant loss of lean mass, which raises concerns about potential long-term impacts on health and physical function. To mitigate this, incorporating higher protein intake and regular RT should be prioritized to help preserve muscle mass and function and optimize weight loss outcomes (Figure 1). If time permits, aerobic training may further enhance body composition and cardiometabolic benefits. Future research should evaluate these strategies in diverse populations of AOM users and explore the contribution of additional lifestyle behavior modification tactics to optimize health and wellness. We believe an integrated approach consisting of pharmacotherapy coupled with holistic lifestyle modification is a powerful strategy for addressing the obesity pandemic and improving overall health and well-being in this growing population.

## Author contributions

Conceptualization: GJG, SSJ.

Literature Search: GJG, MCS.

Writing - Original Draft: GJG.

Writing - Reviewing and Editing: GJG, NVD, JLU, SMA, JGT, HL, MCS, JK, CC, SSJ.

Supervision and Final Approval: GJG, NVD, JLU, SMA, JGT, HL, MCS, JK, CC, SSJ.

## Conflict of interest

GJG, MCS, JK, CC, and SSJ are employees of Medifast Inc. NVD, JLU, SMA, JGT, and HL serve on the Medifast Inc Scientific Advisory Board and received financial support from Medifast during the preparation of this manuscript.

## Funding

The authors reported no funding received for this study.

## Data availability

This narrative review is based on previously published studies and publicly available data. No new data were generated or analyzed specifically for this review. As such, no datasets are available for sharing.
